# Impact of Replacing Soft Drinks with Dairy Products on Micronutrient Intakes of Chinese Preschool Children: A Simulation Study

**DOI:** 10.3390/nu15184071

**Published:** 2023-09-20

**Authors:** Yiding Zhuang, Jia Yin, Fei Han, Jialu You, Ye Ding, Zhixu Wang

**Affiliations:** 1 Department of Maternal, Child and Adolescent Health, School of Public Health, Nanjing Medical University, Nanjing 211166, China; zhuangyiding@stu.njmu.edu.cn (Y.Z.); yinjia0819@stu.njmu.edu.cn (J.Y.); 2 Danone Open Science Research Center for Life-Transforming Nutrition, Shanghai 201204, China; fei.han1@danone.com (F.H.); jialu.you@danone.com (J.Y.)

**Keywords:** Chinese preschool children, soft drinks, dairy products, soymilk, simulation

## Abstract

At present, energy surplus and micronutrient deficiency coexist in preschool children in China. The low intake of dairy products accompanied by an increased consumption of soft drinks in this age group reveals some of the reasons for this phenomenon. The purpose of this study was to evaluate the improvement of key micronutrients in preschool children by quantifying the dietary nutritional gap before and after simulating the use of dairy products instead of equal amounts of soft drinks. In the cross-sectional dietary intake survey of infants and young children in China (2018–2019), 676 preschool children aged 3–6 years were randomly selected. Four days of dietary data were collected through an online diary for simulation. The individual intake of soft drinks was substituted at a corresponding volume by soymilk, cow’s milk, or formulated milk powder for preschool children (FMP-PSC). In these three models, the simulated nutrient intake and nutrient inadequacy or surplus were compared with the actual baseline data of the survey. The results of this study indicated that all three models made the nutrient intakes of this group more in line with the recommendations. For the whole population, the replacement of soymilk improved the intake of zinc (from 4.80 to 4.85 mg/d), potassium (from 824.26 to 836.82 mg/d), vitamin A (from 211.57 to 213.92 μg retinol activity equivalent/d), and vitamin B_9_ (from 115.94 to 122.79 μg dietary folate equivalent/d); the simulation of cow’s milk improved the intake of calcium (from 311.82 to 330.85 mg/d), zinc (from 4.80 to 4.87 mg/d), potassium (from 824.26 to 833.62 mg/d), vitamin A (from 211.57 to 215.12 μg retinol activity equivalent/d), vitamin B_2_ (from 0.53 to 0.54 mg/d), and vitamin B_12_ (from 1.63 to 1.67 μg/d); and the substitution of FMP-PSC improved the intake of calcium (from 311.82 to 332.32 mg/d), iron (from 9.91 to 9.36 mg/d), zinc (from 4.80 to 4.96 mg/d), potassium (from 824.26 to 828.71 mg/d), vitamin A (from 211.57 to 217.93 μg retinol activity equivalent/d), vitamin B_2_ (from 0.53 to 0.54 mg/d), vitamin B_9_ (from 115.94 to 118.80 μg RA dietary folate equivalent/d), and vitamin B_12_ (from 1.63 to 1.70 μg/d). Therefore, correct nutritional information should be provided to parents and preschool children. In addition to changing the consumption behavior of soft drinks, it is also necessary to have a diversified and balanced diet. When necessary, the use of food ingredients or nutritional fortifiers can be encouraged.

## 1. Introduction

The preschool period (3–6 years old) is a crucial stage of children’s growth and development, during which the intake of energy and some key micronutrients (such as calcium, zinc, vitamin A, vitamin B_9_, vitamin D, etc.) is closely related to their health status [1,2]. This period is also a stage where children are prone to form disordered eating behaviors, resulting in imbalanced energy and nutrient intake, which has a profound impact on children’s physical development, cognitive ability, language expression, and motor development [3,4,5]. Therefore, preschool children should follow a balanced diet and avoid the intake of junk food so as to lay the foundation for healthy eating behaviors throughout their lifecycles.

In China, the output of soft drinks has shown a continuous upward trend in the past decade, followed by a huge increase in their consumption [6,7,8]. According to data from the China Health and Nutrition Survey between 2010 and 2012 and the China Food Consumption Survey between 2017 and 2018, the average consumption of soft drinks by urban residents increased from 11.3 g/d to 18.5 g/d [7,8]. A previous study showed that 66.6% of children and adolescents consumed sugar-sweetened drinks, with an average of 2.84 servings per day [9]. In 2019, the number of deaths attributed to sugar-sweetened drinks in China reached 46,633, with an increase of 95% compared with 1990 [10]. With the development of the food industry, sugar-substituted drinks have gained popularity among the public in recent years. They provide the sweetness of sugars without increasing calories, thereby reducing the risk of obesity and dental caries [11,12]. However, they may have adverse effects on children’s health. Some studies have linked the consumption of artificial sweeteners to the risk of obesity, autism, leukemia, and cardiovascular events [13,14].

With the development of the economy, dairy products have become a mature food industry in China. However, unlike soft drinks, the per capita dairy consumption of Chinese residents still has a significant gap compared to the world average [15,16]. According to our research group’s cross-sectional dietary intake survey on infants and young children in China (DSIYC, 2018–2019), 88.31% of preschool children did not meet the recommended 350 g per day of dairy products [17]. As an excellent source of calcium, phosphorus, magnesium, vitamin A, B vitamins, and vitamin D, dairy products are recognized worldwide as an ideal dietary component to improve the nutritional status of preschool children [18]. The low level of dairy consumption among preschool children is a direct cause of an inadequate intake of calcium, vitamin A, and other micronutrients, which is a cause for concern. Taking calcium as an example, the proportion of Chinese preschool children whose calcium intake was lower than the estimated average requirement (EAR) reached 91.57% [17].

Therefore, with the premise that household food costs can be borne, reducing the consumption of soft drinks and increasing the intake of dairy products may be a breakthrough to improve the nutritional status of preschool children in China. Although several cross-sectional surveys were conducted to measure the consumption of soft drinks and dairy products among preschool children in China [19,20], there is no relevant study to quantify the nutritional gap caused by this measure. Based on the data from the DSIYC from 2018 to 2019, we conducted a simulation study. Given that micronutrient fortification of dairy products is allowed for preschool children in China [21], in addition to cow’s milk, we also chose formulated milk powder for preschool children (FMP-PSC) to replace the individual intake of soft drinks with a matching volume. Moreover, soymilk as a Chinese characteristic food was also selected in the simulation. We hypothesized that the improvement of key micronutrients of preschool children would be observed when soymilk, cow’s milk, and FMP-PSC are used to simulate each model, and FMP-PSC has the greatest improvement.

## 2. Materials and Methods

### 2.1. Study Sample

Analyses were carried out utilizing data from the DSIYC from 2018 to 2019. The design of the DSIYC covered 11 provinces and 2 municipalities in mainland China based on geographic positions, local economic levels, and annual birth rate. They included Guangdong, Liaoning, Sichuan, Hebei, Yunnan, Henan, Fujian, Hubei, Zhejiang, Anhui, Jiangsu, Beijing, and Shanghai. The cities were then selected to conduct the survey based on the urban and rural areas in different regions. Preschool children were finally randomly selected from each city on the basis of the information provided by the Maternal and Child Health Center. Healthy children aged between 36 and 72 months, born full-term as singletons were eligible for inclusion in this study. Those with a birth weight <2500 g or >4000 g or with parents with limited cognitive abilities who were unable to answer questions or have poor emotions and were unable to cooperate, were excluded. Data were obtained from a total of 678 children, of which 2 were excluded due to a lack of meals, and the data of 676 children were ultimately included in the analysis, including 224 children aged 3–4 years, 226 children aged 4–5 years, and 226 children aged 5–6 years.

### 2.2. Dietary Data Collection

The collection of four-day dietary data for preschool children, using an online diary with a food atlas to improve the accuracy of estimation, has been described previously [17]. The trade names of soft drinks and dietary supplements were also recorded during the survey. Based on the consumption characteristics of soft drinks for preschool children, the soft drinks in this survey were divided into 7 categories: carbonated drinks, fruit and vegetable drinks, protein drinks, tea drinks, botanical drinks, flavored drinks, and solid drinks (Table 1).

### 2.3. Simulation Replacement Models and Analysis

In this study, 3 simulations were conducted in which the individual intake of soft drinks was replaced by soymilk (model 1), cow’s milk (model 2), and FMP-PSC (model 3) at a matching volume. The daily nutrient intakes and nutrient inadequacy before and after the simulation were calculated. Specifically, the dietary data were converted and calculated into the corresponding energy and nutrient contents according to the Chinese Food Composition Table (6th edition) [22]. The nutrient information labeled on soft drinks and dietary supplements was then calculated together with the nutrient content consumed in the diet. Further evaluation of daily nutrient intake was conducted on the 2013 Chinese Dietary Reference Intakes (DRIs) [23]. The indicator for evaluating an inadequate potassium intake was adequate intake (AI), while an inadequate intake of other micronutrients referred to the intake below their estimated average requirements (EAR). Table 2 presents the nutritional composition of soymilk, cow’s milk, and FMP-PSC used for the simulation.

### 2.4. Statistical Analysis

The data analyses were performed using SPSS software package version 27.0 (IBM, New York, NY, USA). After testing, almost all the data were of non-normal distribution. Therefore, the continuous variable data were presented as median (*P*_25_, *P*_75_). Categorical variable data were displayed as frequency (n) and percentage (%). The differences in soft drink intake among different age groups were compared by a Kruskal–Wallis H test or a Chi-square test. A Wilcoxon signed-rank test was applied to compare the energy intake before and after modeling, and the Kruskal–Wallis H test was used to assess the energy changes after simulating different models. A Wilcoxon signed-rank test and a McNemar test were applied to test the differences in nutrient intake and the proportion of preschool children with inadequate or excessive nutrient intake before and after modeling. A value of *p* < 0.05 was considered to be statistically significant.

## 3. Results

### 3.1. The Intake of Soft Drinks before Simulation

The intake distribution of soft drinks before the simulation can be found in Table 3. Within 4 days, 178 out of 676 preschool children aged 37–72 months consumed soft drinks, accounting for 26.33%, with a median intake of 40.0 g/d. After grouping by age, it was found that as children grew older, the proportion of soft drinks consumed increased significantly, but there was no significant difference in total intake among different age groups. The most common soft drinks consumed were dairy drinks; 19.67% of preschool children consumed dairy drinks within 4 days, with a median daily intake of 27.5 g. Similar to the total intake of soft drinks, the proportion of preschool children who consumed dairy drinks significantly increased with age, but there was no significant difference in the intake of dairy drinks among different age groups.

### 3.2. Total Energy and Macronutrient Intakes after Simulation

After the intake of soft drinks was substituted by the same volume of soymilk (model 1), cow’s milk (model 2), and FMP-PSC (model 3), the total energy intake of children in all three models was significantly decreased when compared with the reported data, especially in the substitution of soymilk. Furthermore, it was found that the differences in energy changes among the three models were statistically significant (Table 4). As shown in Table 5, the changes in carbohydrate intake in each model were similar to those in energy, which caused a slight increase in the proportion of children with inadequate carbohydrate intake, particularly with soymilk. The intake of protein and fat in all three models increased slightly compared to the reported data, and the proportion of children with inadequate protein intake did not change much.

### 3.3. Mineral Intakes after Simulation

As shown in Table 6, the substitution of soymilk (model 1) did not affect the calcium intake of children, but the simulation of dairy products increased the calcium intake and reduced the number of children with inadequate calcium intake in the total population, especially with the replacement of FMP-PSC (model 3). The trend of changes in iron and iodine intake was similar. Compared with the reported data, the intake of these two minerals significantly increased in all three models. Especially with the replacement of FMP-PSC (model 3), the median intake of iron and iodine reached 9.36 mg/d and 45.23 µg/d, respectively, and the proportion of children with inadequate iron intake decreased to some extent. In all three models, the intake of zinc increased and the proportion of children with inadequate zinc intake decreased. The most obvious change was observed in the replacement of FMP-PSC (model 3), with the proportion of children with inadequate zinc intake dropping from 37.57% to 34.47%. The simulation also increased children’s potassium intake; most notable was the replacement with soybean milk (model 1), which reduced the proportion of children with inadequate potassium intake, dropping from 73.96% to 71.30%.

### 3.4. Vitamin Intakes after Simulation

As shown in Table 7, the intake of vitamin B_1_, vitamin B_3_, and vitamin B_6_ increased in all three models, with the most significant changes observed in the FMP-PSC replacement (model 3). However, none of these models resulted in a significant change in the proportion of children with inadequate intake of these three B vitamins. Compared with the reported data, the intake of vitamin A and vitamin B_12_ in children increased after the simulation, and the proportion of children with an inadequate intake of these two vitamins decreased, especially after the dairy simulation. FMP-PSC showed the strongest improvement in the inadequate intake of vitamin A and vitamin B_12_. The vitamin B_2_ intake was significantly decreased after the replacement of soymilk (model 1), but after the simulation with dairy products, its intake was significantly increased and the proportion of children with an inadequate intake thereof was decreased. After the simulation, children’s intake of vitamin B_9_ was significantly improved, especially when soymilk was replaced. In the dairy product simulation, FMP-PSC improved the inadequate vitamin B_9_ intake better than cow’s milk. All three models resulted in a decrease in vitamin C intake and an increase in the proportion of children with inadequate intake thereof. Although the vitamin D intake was significantly increased after the replacement with FMP-PSC, the improvement of an inadequate intake of vitamin D was limited in all three models.

## 4. Discussion

Drinks are an important part of one’s overall dietary intake. With ongoing concerns about obesity and poor nutrition among preschool children, the consumption of soft drinks in this age group has emerged as an important public health concern [24,25]. This simulation study, which was based on the dietary data of 676 preschool children aged 3-6 years in China, innovatively selected soymilk, cow’s milk, and FMP-PSC to replace the individual intake of soft drinks at a matching volume. The dietary nutritional gap between pre- and post-simulation was then quantified to evaluate the improvement of key micronutrients in preschool children. So far, very limited information on this aspect of preschool children is available. The findings of this study indicated that all three models brought nutrient intakes of preschool children more in line with the recommendations. For the entire population, the replacement of soymilk improved the intake of zinc, potassium, vitamin A, and vitamin B_9_, while the simulation of dairy products, including cow’s milk and FMP-PSC, improved the intake of calcium, zinc, potassium, vitamin A, vitamin B_2_, and Vitamin B_12_. In addition, the replacement of FMP-PSC also improved the intake of iron and vitamin B_9_.

Dietary habits are established during the preschool stage, and replacing water with soft drinks is an unhealthy eating behavior that needs to be corrected in a timely manner. In this study, dairy drinks were the most frequently consumed soft drinks by preschool children. Dairy drinks may contain certain amounts of protein, vitamins, and minerals, but their nutritional value is far inferior to dairy products [26]. Studies have demonstrated that the dietary habits and feeding strategies of parents are the most significant factors in determining their children’s eating behavior and food choices [27]. Numerous Chinese parents tend to overlook the nutritional composition table while buying dairy products or drinks, and a few parents even hold the belief that dairy products and dairy drinks have equal nutritional value [28]. Thus, it is imperative to reinforce health education for parents to aid them in expanding their nutritional knowledge, adopting scientific consumption concepts, and correctly evaluating the nutritional value of drinks. This will help to restrict the consumption of soft drinks among preschool children.

Currently, over half of the soft drinks available on the Chinese market are sugar-sweetened drinks, with the majority having a sugar content ranging from 8% to 11% [29]. As expected, following the simulation, it was observed that the total energy intake of children in all three models was significantly reduced in comparison to the reported data, particularly with the substitution of soymilk. Upon further analysis of the changes in macronutrients, it was revealed that this decrease in energy was, in fact, due to a reduction in carbohydrate intake. The consumption of sugar-sweetened drinks might lead to excessive energy intake, thereby contributing to the growing problem of childhood obesity in China. Sugar-sweetened drinks also increase the risk of dental cavities and developing type 2 diabetes, heart disease, and gout among children and adolescents [30,31,32]. Soymilk is produced by soaking soybeans and grinding them with water. Half of the carbohydrates in soybeans are oligosaccharides that the human body cannot digest and absorb, which assists in maintaining stable blood sugar levels and gastrointestinal health in children [33]. The form in which carbohydrates exist in dairy products is lactose. The FMP-PSC utilized in this study’s simulation did not contain added sucrose. Lactose is a disaccharide that consists of glucose and galactose. During the growth and development of infants and young children, lactose not only provides them with energy but also participates in the development process of their brains [33].

It is crucial to ensure adequate calcium intake during the preschool period, and supplementing dietary calcium intake in the diet can significantly enhance bone mineral density, particularly in children with low baseline calcium intakes, which is currently the case in China [17]. In this study, it was found that the substitution of soymilk did not affect children’s calcium intake. This could be easily explained by the fact that the most commonly consumed soft drinks among preschool children in this study were dairy drinks, most of which contained low concentrations of calcium. Additionally, the calcium concentration of soymilk used for the simulation was also relatively low. There is no doubt that dairy products provide the greatest amount of calcium in our diet. A previous laboratory feeding study confirmed that an increase in the intake of sugar-sweetened drinks among children aged 3 to 7 years leads to a corresponding decrease in milk intake, thereby reducing their calcium intake [34]. In our study, we chose cow’s milk and FMP-PSC to replace the individual intake of soft drinks with a matching volume, and our findings showed that the simulation of dairy products did indeed increase the calcium intake and reduce the number of children with inadequate calcium intake in the total population, particularly when replacing individual intake of soft drinks with FMP-PSC.

Infants are routinely supplemented with vitamin A supplements in numerous regions of China. However, their duration is relatively short, coupled with insufficient levels of vitamin A and ꞵ-carotene in their daily diet, and they also have low efficiency in converting ꞵ-carotene into vitamin A in the body, resulting in a high proportion of vitamin A deficiency (VAD) or marginal VAD in Chinese children [35], which will lead to visual dysfunction, growth and development disorders, and low immune function [33]. In our study, compared to the reported data, the intake of vitamin A in children increased and the proportion of children with inadequate intake thereof decreased in all three models. The FMP-PSC model showed the strongest improvement, which was similar to the results of our previous simulation study [17]. When increasing the amount of cow’s milk or FMP-PSC to ensure that each child’s dairy intake reached the recommended amount (350 g/day), FMP-PSC had a more significant effect on improving vitamin A intake compared to cow’s milk [17].

There are two interesting findings in our study. Firstly, when compared to the reported data, it was found that children’s intake of vitamin C decreased in all three models. The reason for this may be that vitamin C has always been a popular element emphasized by manufacturers in the history of beverage development, aiming to attract consumers [36]. In fact, both before and after modeling, close to half of the population suffered from inadequate vitamin C intake. A balanced diet and the intake of fruits and vegetables hold greater significance in enhancing the population’s vitamin C intake [33]. Secondly, modeling did not reduce the prevalence of inadequate vitamin D intakes. Very few foods have naturally occurring vitamin D [33]. Fortified foods are the primary source of vitamin D in the Chinese diet [33]. In our study, the replacement of FMP-PSC fortified with vitamin D solely increased its intake, while the proportion of inadequate intake in the entire population remained unchanged. A possible explanation is that vitamin D intake at baseline is considerably lower than the recommended amount for a large portion of the population. Therefore, it is crucial to implement comprehensive nutritional interventions and provide healthy lifestyle education to improve the status of vitamin D [37].

This study has some limitations to consider. Firstly, the nutritional gaps of preschool children were only evaluated by replacing soft drinks; however, there are many other potential sources of extra sugar consumption in real life, such as snacks and desserts, which may be an emerging concern that demands close attention in the future. Secondly, this study focused on the impact of drink simulation on the population, thus the EAR and AI were utilized to evaluate nutrient adequacy [23]; however, on the basis of personal nutritional counseling, the recommended nutrient intake (RNI) should be adopted [23], which may result in a larger nutritional gap that requires additional dairy products or other nutritional interventions to ensure adequate nutrient intake. Lastly, some nutrients do not entirely come from food, soft drinks, and dietary supplements. For example, plain water contains certain minerals, and the status of vitamin D is intricately associated with one’s lifestyle. In future research, these factors should be comprehensively considered to better evaluate nutrient status.

## 5. Conclusions

This simulation evaluated the effect of replacing soft drinks with soymilk, cow’s milk, and FMP-PSC on the dietary nutrition improvement of Chinese preschool children. The results suggest that we should pay more attention to the lack of key micronutrients, abuse of soft drinks, and low consumption of dairy products in this group. In order to achieve the optimal nutritional intake for preschool children, relevant policies should be implemented to protect children from the harmful effects of soft drink marketing. Correct nutritional information should also be provided to parents and preschool children to ensure a diversified and balanced diet. When necessary, the use of food ingredients or nutritional fortifiers can be encouraged.

## Figures and Tables

**Table 1:** Description of the soft drink categories.

Categories	Definitions	Examples
Carbonated drinks	This refers to drinks filled with carbon dioxide gas under certain conditions.	Coca Cola, Sprite, Fanta
Fruit and vegetable drinks	This refers to processed drinks made by mixing fruit and/or vegetable juice with water, sugar (or sweeteners), acidulants, etc.	Minute Maid, Nongfu orchard 30% mixed fruit juice, Huiyuan fruit and vegetable juice
Protein drinks	This refers to processed or fermented drinks made from dairy products, seeds, or fruits of plants with a certain amount of protein, with the addition of water, sugar (or sweeteners), acidulants, etc.	Yakult, Wahaha AD calcium milk, Lulu almond juice
Tea drinks	This refers to processed drinks made from tea extract, tea powder, or tea concentrated liquid, with added water, sugar (or sweeteners), acidulants, spices, etc.	Master Kong tea beverage, Uni-President Assam milk tea beverage, Nongfu Spring fruit-flavored tea beverage
Botanical drinks	This refers to processed or fermented drinks made from plants or plant extracts other than fruits, vegetables, tea, and coffee.	Chinese herb beverage, honeysuckle beverage, Master Kang’s sour plum soup
Flavored drinks	This refers to processed drinks adjusting flavor through edible essences (or spices), sugar (or sweeteners), acidulants, etc.	Nongfu Spring fruit-flavored beverage, Paldo Pororo flavored beverage, Master Kong flavored beverage
Solid drinks	This refers to processed products made from food ingredients and food additives into solid forms such as powders, granules, or blocks for brewing.	Tang, Ovaltine, Cola Cao

**Table 2:** Nutritional composition per 100 g of soymilk, cow’s milk, and FMP-PSC used for the simulation.

Parameters	Soymilk	Cow’s Milk	FMP-PSC
Energy (kcal)	31	54	456.8
Carbohydrate (g)	1.2	3.4	44.7
Protein (g)	3	3	19.4
Fat (g)	1.6	3.2	20.7
Calcium (mg)	5	104	867
Iron (mg)	0.4	0.3	8.2
Zinc (mg)	0.28	0.42	7.74
Iodine (µg)	2.1	1.9	41.9
Potassium (mg)	117	109	462
Vitamin A (μg RE)	15	24	276
Vitamin B_1_ (mg)	0.02	0.03	0.35
Vitamin B_2_ (mg)	0.02	0.14	0.77
Vitamin B_3_ (mg)	0.14	0.1	2.48
Vitamin B_6_ (mg)	0.019	0.036	0.27
Vitamin B_9_ (μg DFE)	39.4	5	100
Vitamin B_12_ (μg)	0.1	0.2	2.7
Vitamin C (mg)	/	1	26.3
Vitamin D (μg)	/	/	4.7

FMP-PSC: formulated milk powder for preschool children (Aptamil); RE: retinol equivalent; DFE: dietary folate equivalent.

**Table 3:** The intake of soft drinks before simulation.

Parameters		All (n = 676)	37~48 Months (n = 224)	49~60 Months (n = 226)	61~72 Months (n = 226)	*P* ^Δ^
Soft drinks	N (%)	178 (26.33%)	51 (22.77%)	53 (23.45%)	74 (32.74%)	0.027
	g/d *	40.0 (25.0, 75.0)	37.5 (25.0, 55.0)	45.0 (25.0, 75.0)	42.0 (25.0, 79.0)	0.414
Dairy drinks	N (%)	133 (19.67%)	36 (16.07%)	39 (17.26%)	58 (25.66%)	0.020
	g/d *	27.5 (25.0, 50.0)	27.5 (25.0, 50.0)	50.0 (25.0, 55.0)	27.5 (25.0, 50.0)	0.314

* Data were expressed in median (*P*_25_, *P*_75_) and only represented the situation of children who consumed soft drinks or dairy drinks. ^Δ^ *P* was assessed using the Chi-square test or Kruskal–Wallis H test for the differences in soft drink intake among different age groups.

**Table 4:** Total energy intake after simulation (n = 676) (kcal/d).

Groups	Total Energy (Median (*P*_25_, *P*_75_))	Changes of Energy (Median (Min, Max))	*P* ^Δ^
Before simulation	993.99 (765.65, 1256.99)	/	/
Model 1	982.78 (765.65, 1234.53) ^a^	0.00 (−463.50, 63.28)	<0.001
Model 2	987.58 (768.02, 1235.99) ^bd^	0.00 (−429.00, 141.48)	
Model 3	992.84 (769.05, 1242.75) ^cef^	0.00 (−406.70, 192.01)	

A Wilcoxon signed-rank test was used to compare the differences in energy intake before and after modeling, respectively. ^a^ Model 1 compared to before simulation, *p* < 0.05; ^b^ model 2 compared to before simulation, *p* < 0.05; ^c^ model 3 compared to before simulation, *p* < 0.05; ^d^ model 2 compared to model 1, *p* < 0.05; ^e^ model 3 compared to model 1, *p* < 0.05; ^f^ model 3 compared to model 2, *p* < 0.05. Model 1: The intake of soft drinks was replaced by soymilk at the same volume. Model 2: The intake of soft drinks was replaced by cow’s milk at the same volume. Model 3: The intake of soft drinks was replaced by FMP-PSC at the same volume. ^Δ^ *P* was assessed using the Kruskal–Wallis H test for the energy changes after simulating different models.

**Table 5:** Macronutrient intakes after simulation (n = 676).

Macronutrients	Groups	Median (*P*_25_, *P*_75_)	N (%)
Carbohydrate (g/d)	Before simulation	109.81 (83.05, 145.60)	388 (57.40%)
	Model 1	109.38 (82.86, 142.33) ^a^	398 (58.88%) ^a^
	Model 2	109.59 (83.05, 143.12) ^bd^	396 (58.58%) ^b^
	Model 3	110.09 (83.18, 143.39) ^cef^	390 (57.69%) ^ef^
Protein (g/d)	Before simulation	32.65 (25.27, 43.05)	161 (23.82%)
	Model 1	33.09 (25.64, 43.68) ^a^	158 (23.37%)
	Model 2	33.09 (25.64, 43.68) ^b^	158 (23.37%)
	Model 3	33.09 (25.64, 43.68) ^cef^	158 (23.37%)
Fat (g/d)	Before simulation	37.87 (29.81, 48.28)	/
	Model 1	37.92 (30.10, 48.98) ^a^	/
	Model 2	38.26 (30.16, 49.42) ^bd^	/
	Model 3	38.22 (30.16, 49.41) ^cef^	/

A Wilcoxon signed-rank test and a McNemar test were used to compare the differences in nutrient intake and the changes in the proportion of preschool children with inadequate nutrient intake before and after modeling, respectively. ^a^ Model 1 compared to before simulation, *p* < 0.05; ^b^ model 2 compared to before simulation, *p* < 0.05; ^c^ model 3 compared to before simulation, *p* < 0.05; ^d^ model 2 compared to model 1, *p* < 0.05; ^e^ model 3 compared to model 1, *p* < 0.05; ^f^ model 3 compared to model 2, *p* < 0.05. Model 1: The intake of soft drinks was replaced by soymilk at the same volume. Model 2: The intake of soft drinks was replaced by cow’s milk at the same volume. Model 3: The intake of soft drinks was replaced by FMP-PSC at the same volume.

**Table 6:** Mineral intakes after simulation (n = 676).

Minerals	Groups	Median (*P*_25_, *P*_75_)	N (%)
Calcium (mg/d)	Before simulation	311.82 (214.98, 425.66)	619 (91.57%)
	Model 1	311.66 (211.35, 424.27) ^a^	620 (91.72%)
	Model 2	330.85 (226.59, 445.26) ^bd^	610 (90.24%) ^bd^
	Model 3	332.32 (228.27, 451.43) ^cef^	606 (89.64%) ^ce^
Iron (mg/d)	Before simulation	9.19 (6.97, 12.02)	143 (21.15%)
	Model 1	9.26 (6.98, 12.05) ^a^	140 (20.71%)
	Model 2	9.25 (6.98, 12.03) ^bd^	140 (20.71%)
	Model 3	9.36 (7.05, 12.23) ^cef^	136 (20.12%) ^c^
Zinc (mg/d)	Before simulation	4.80 (3.59, 6.36)	254 (37.57%)
	Model 1	4.85 (3.62, 6.39) ^a^	248 (36.69%) ^a^
	Model 2	4.87 (3.63, 6.43) ^bd^	243 (35.95%) ^b^
	Model 3	4.96 (3.66, 6.63) ^cef^	233 (34.47%) ^cef^
Iodine (µg/d)	Before simulation	43.22 (16.10, 590.62)	394 (58.28%)
	Model 1	43.68 (16.27, 590.75) ^a^	394 (58.28%)
	Model 2	43.58 (16.19, 590.74) ^bd^	394 (58.28%)
	Model 3	45.23 (16.75, 590.79) ^cef^	391 (57.84%)
Potassium (mg/d)	Before simulation	824.26 (606.15, 1134.74)	500 (73.96%)
	Model 1	836.82 (618.19, 1161.51) ^a^	482 (71.30%) ^a^
	Model 2	833.62 (615.88, 1160.93) ^bd^	484 (71.60%) ^b^
	Model 3	828.71 (608.95, 1151.43) ^cef^	490 (72.49%) ^cef^

The intakes of calcium, iron, zinc, and iodine below the estimated average requirement were considered inadequate, while the adequate intake was used to assess potassium inadequacy. A Wilcoxon signed-rank test and a McNemar test were used to compare the differences in nutrient intake and the changes in the proportion of preschool children with inadequate nutrient intake before and after modeling, respectively. ^a^ Model 1 compared to before simulation, *p* < 0.05; ^b^ model 2 compared to before simulation, *p* < 0.05; ^c^ model 3 compared to before simulation, *p* < 0.05; ^d^ model 2 compared to model 1, *p* < 0.05; ^e^ model 3 compared to model 1, *p* < 0.05; ^f^ model 3 compared to model 2, *p* < 0.05. Model 1: The intake of soft drinks was replaced by soymilk at the same volume. Model 2: The intake of soft drinks was replaced by cow’s milk at the same volume. Model 3: The intake of soft drinks was replaced by FMP-PSC at the same volume.

**Table 7:** Vitamin intakes after simulation (n = 676).

Vitamins	Groups	Median (*P*_25_, *P*_75_)	N (%)
Vitamin A (µg RAE/d)	Before simulation	211.57 (148.37, 293.54)	429 (63.46%)
	Model 1	213.92 (150.32, 296.81) ^a^	420 (62.13%) ^a^
	Model 2	215.12 (151.00, 298.62) ^bd^	415 (61.39%) ^b^
	Model 3	217.93 (151.85, 301.30) ^cef^	405 (59.91%) ^cef^
Vitamin B_1_ (mg/d)	Before simulation	0.33 (0.24, 0.46)	596 (88.17%)
	Model 1	0.33 (0.25, 0.46) ^a^	596 (88.17%)
	Model 2	0.34 (0.25, 0.46) ^bd^	595 (88.02%)
	Model 3	0.34 (0.25, 0.47) ^cef^	593 (87.72%)
Vitamin B_2_ (mg/d)	Before simulation	0.53 (0.36, 0.73)	367 (54.29%)
	Model 1	0.52 (0.36, 0.73) ^a^	369 (54.59%)
	Model 2	0.54 (0.37, 0.75) ^bd^	355 (52.51%) ^bd^
	Model 3	0.54 (0.36, 0.75) ^cef^	357 (52.81%) ^ce^
Vitamin B_3_ (mg NE/d)	Before simulation	6.78 (5.12, 9.45)	249 (36.83%)
	Model 1	6.80 (5.13, 9.49) ^a^	249 (36.83%)
	Model 2	6.80 (5.13, 9.47) ^bd^	249 (36.83%)
	Model 3	6.86 (5.16, 9.56) ^cef^	247 (36.54%)
Vitamin B_6_ (mg/d)	Before simulation	0.71 (0.50, 0.93)	213 (31.51%)
	Model 1	0.71 (0.51, 0.94) ^a^	212 (31.36%)
	Model 2	0.71 (0.51, 0.94) ^bd^	212 (31.36%)
	Model 3	0.71 (0.51, 0.94) ^cef^	210 (31.07%)
Vitamin B_9_ (µg DFE/d)	Before simulation	115.94 (84.86, 156.99)	465 (68.79%)
	Model 1	122.79 (90.19, 169.04) ^a^	437 (64.64%) ^a^
	Model 2	116.65 (85.01, 158.90) ^bd^	462 (68.34%)
	Model 3	118.80 (86.21, 160.51) ^cef^	458 (67.75%) ^ce^
Vitamin B_12_ (µg/d)	Before simulation	1.63 (1.04, 2.54)	139 (20.56%)
	Model 1	1.66 (1.04, 2.58) ^a^	136 (20.12%)
	Model 2	1.67 (1.05, 2.58) ^bd^	133 (19.67%) ^b^
	Model 3	1.70 (1.07, 2.62) ^cef^	125 (18.49%) ^cef^
Vitamin C (mg/d)	Before simulation	42.47 (22.56, 69.87)	302 (44.67%)
	Model 1	39.29 (21.50, 66.18) ^a^	326 (48.22%) ^a^
	Model 2	39.30 (21.69, 66.57) ^d^	326 (48.22%) ^b^
	Model 3	39.72 (21.91, 67.55) ^ef^	323 (47.78%) ^c^
Vitamin D (µg/d)	Before simulation	0.00 (0.00, 1.82)	666 (98.52%)
	Model 1	0.00 (0.00, 1.82)	666 (98.52%)
	Model 2	0.00 (0.00, 1.82)	666 (98.52%)
	Model 3	0.24 (0.00, 1.91) ^cef^	666 (98.52%)

RAE, retinol activity equivalent; NE, nicotinic acid equivalent; DFE, dietary folate equivalent. The intakes of vitamins below the estimated average requirement were considered inadequate. A Wilcoxon signed-rank test and a McNemar test were used to compare the differences in nutrient intake and the changes in the proportion of preschool children with inadequate nutrient intake before and after modeling, respectively. ^a^ Model 1 compared to before simulation, *p* < 0.05; ^b^ model 2 compared to before simulation, *p* < 0.05; ^c^ model 3 compared to before simulation, *p* < 0.05; ^d^ model 2 compared to model 1, *p* < 0.05; ^e^ model 3 compared to model 1, *p* < 0.05; ^f^ model 3 compared to model 2, *p* < 0.05. Model 1: The intake of soft drinks was replaced by soymilk at the same volume. Model 2: The intake of soft drinks was replaced by cow’s milk at the same volume. Model 3: The intake of soft drinks was replaced by FMP-PSC at the same volume.

## Data Availability

The data presented in this study are available on request from the corresponding author.
